# Are people’s health care needs better met when primary care is strong? A synthesis of the results of the QUALICOPC study in 34 countries

**DOI:** 10.1017/S1463423619000434

**Published:** 2019-07-01

**Authors:** Willemijn L.A. Schäfer, Wienke G.W. Boerma, Michael J. van den Berg, Jan De Maeseneer, Sabina De Rosis, Jens Detollenaere, Stefan Greß, Stephanie Heinemann, Tessa van Loenen, Anna Maria Murante, Danica R. Pavlič, Chiara Seghieri, Milena Vainieri, Sara Willems, Peter P. Groenewegen

**Affiliations:** 1 Department of Surgery, Northwestern University, Feinberg School of Medicine, 633 N. St Clair Street, Chicago, IL 60611, USA; 2 NIVEL – Netherlands Institute for Health Services Research, PO box 1568, 3500BN Utrecht, The Netherlands; 3 Amsterdam UMC, University of Amsterdam, Department of Public Health, Amsterdam Public Health Research Institute, 22660, 1100 DD, Amsterdam; 4 Department of Public Health and Primary Care, Ghent University, Corneel Heymanslaan 10, Ghent, Belgium; 5 Scuola Superiore Sant’Anna, Institute of Management, Laboratorio Management e Sanità, piazza Martiti della Libertà 33, Pisa 56127, Italy; 6 KCE – Belgian Health Care Knowledge Centre, Kruidtuinlaan 55, 1000 Brussels, Belgium; 7 Department of Nursing and Health Sciences, University of Applied Sciences Fulda, Leipziger Str. 123, 36037 Fulda, Germany; 8 Department of General Practice, University Medical Center Göttingen, Humboldtallee 38, 37073 Göttingen, Germany; 9 Pharos – Centre of Expertise on Health Disparities, PO box 13318, 3507 LH Utrecht, The Netherlands; 10 Department of Family Medicine, University of Ljubljana, Poljanski nasip 58, 1000 Ljubljana, Slovenia; 11 Department of Sociology and Department of Human Geography, Utrecht University, P.O. Box 80.115, 3508 TC Utrecht, The Netherlands

**Keywords:** Health Services Research, primary care

## Abstract

**Aim:**

This article synthesises the results of a large international study on primary care (PC), the QUALICOPC study.

**Background:**

Since the Alma Ata Declaration, strengthening PC has been high on the policy agenda. PC is associated with positive health outcomes, but it is unclear how care processes and structures relate to patient experiences.

**Methods:**

Survey data were collected during 2011–2013 from approximately 7000 PC physicians and 70 000 patients in 34, mainly European, countries. The data on the patients are linked to data on the PC physicians within each country and analysed using multilevel modelling.

**Findings:**

Patients had more positive experiences when their PC physician provided a broader range of services. However, a broader range of services is also associated with higher rates of hospitalisations for uncontrolled diabetes, but rates of avoidable diabetes-related hospitalisations were lower in countries where patients had a continuous relationship with PC physicians. Additionally, patients with a long-term relationship with their PC physician were less likely to attend the emergency department. Capitation payment was associated with more positive patient experiences. Mono- and multidisciplinary co-location was related to improved processes in PC, but the experiences of patients visiting multidisciplinary practices were less positive. A stronger national PC structure and higher overall health care expenditures are related to more favourable patient experiences for continuity and comprehensiveness. The study also revealed inequities: patients with a migration background reported less positive experiences. People with lower incomes more often postponed PC visits for financial reasons. Comprehensive and accessible care processes are related to less postponement of care.

**Conclusions:**

The study revealed room for improvement related to patient-reported experiences and highlighted the importance of core PC characteristics including a continuous doctor–patient relationship as well as a broad range of services offered by PC physicians.

## Introduction

Effective health systems must be accessible and responsive to the health and social needs of people. Particularly in developed countries, health needs have changed considerably while health systems have not adjusted their services adequately. Specifically, chronic conditions and multimorbidity, which have come along with the aging of populations, are challenging health systems. Curative care continues to be provided predominantly in fragmented single-disease approaches, whereas large-scale efforts to systematically identify groups at risk and develop programmes for behavioural change to reduce or prevent chronic diseases are still scarce. In addition to the increases in costs due to developments in medical technology, the absence of lifestyle medicine and goal-oriented care could be an important reason why countries fail to curb the trend of rising health expenditures (De Maeseneer and Boeckxstaens, 2012).

Furthermore, health systems have difficulty reaching deprived or marginalised groups, thus not addressing health inequalities. Lack of health coverage and high out-of-pocket payments, often coupled with poor health literacy and social problems, can keep these groups from seeking necessary care or adhering to a treatment plan (European Commission Expert Panel on effective ways of investing in Health, 2014; 2016).

Previous studies show that strong primary care (PC), rooted in the community, can make health systems more responsive and effective (Starfield *et al*., 2005; World Health Organization, 2008). Key characteristics of strong PC are equity in access and continuity of care over time, availability of a comprehensive set of high-quality curative and preventive services in conformity to people’s needs, and oversight and coordination of care provided across levels and types of services (Starfield, 1994; Kringos *et al*., 2010). National policies should therefore promote the integration of services and organise systems around the needs and goals of people rather than around diseases. Such care arrangements, where patients and their needs are known and are approached actively, where a balanced combination of curative and preventive care is offered and where information is available to coordinate complex care, must be located at the primary level (World Health Organization, 2008).

Since the Alma Ata Declaration in 1978, strengthening PC has been a high priority on national and international policy agendas (World Health Organization, 1978). ‘Integrated people-centred health care systems’, as recently promoted by the World Health Organisation to achieve universal health coverage, can more easily be realised if based on well-developed and strong PC (Pettigrew *et al*., 2015; Stigler et al., 2016; World Health Organisation, 2016). Recently, the Alma Ata Declaration was reaffirmed through the 2018 Declaration of Astana (Declaration of Astana, 2018).

The evidence base for the added value of strong community-based PC is growing, but several gaps remain. Outcomes from international studies are sometimes hard to generalise due to a selective number of countries included or because of a high level of aggregation. Apart from this, experiences of patients and issues related to equity have rarely been investigated (Kringos *et al*., 2010). The current state of knowledge allows the conclusion that countries with strong PC systems perform better in a number of dimensions, but less is known about the underlying mechanisms.

The QUALICOPC (Quality and Costs of Primary Care in Europe) study aimed to open up this black box and shed light on the structural and process features that contribute to better and more equitable patient outcomes and system performance (Schäfer *et al*., 2011). Using data from almost 70 000 patients and over 7000 PC physicians in 34 countries, this study is unique in terms of scale and granularity. The study took both the perspectives of patients and PC physicians in considering the benefits of strong PC.

This article provides a synthesis of the results of this extensive study to inform about the benefits and opportunities of strong PC. Our synthesis is based on a large number of articles focusing on specific aspects of PC and based on the data collected in the QUALICOPC study. As a consequence, this synthesising article contains little detail on methods and analysis applied in the contributing articles. The added value of this synthesis, however, is in the comprehensiveness of the overview of results of this large and unique study.

After a brief summary of the methodology of the QUALICOPC study, we present the results along Donabedian’s well-known model which includes structure, process, and outcomes (Donabedian, 1988). The results section has four parts which begin at the patient level and move successively higher. First, we start from the outcomes of PC in terms of patient enablement (a patient-reported outcome), the patient-perceived improvement potential, patient-reported equity in treatment and access, and the efficiency of PC. In the second part of the results, we explain how these outcomes were found to be related to processes in PC practices. Based on PC physicians’ reports on the processes, we discuss continuity, coordination, comprehensiveness of care and community orientation, and the associations with the patient experiences and efficiency, that is, the outcomes of PC. Third, we connect the outcomes and care processes to the structure and organisation of the PC practices. Fourth, we take into account national structures of PC (economic conditions, workforce development, and governance) (Kringos *et al*., 2010) and health expenditure and look for associations between these structures and the processes and outcomes within PC. In the discussion, we will highlight the lessons that can be drawn from the study.

## Methods

### Drafting this paper

For this article, we synthesised the results of 21 papers published in peer-reviewed journals, based on the international QUALICOPC dataset. The synthesis has been undertaken by researchers of the international coordinating team that conducted the study, as represented by the authors of this article.

### QUALICOPC survey

Data were collected between 2011 and 2013 among 7183 PC physicians and 69,201 patients in 31 European countries (EU 27 – except France – plus Iceland, Norway, the Republic of Macedonia, Turkey, and Switzerland) and three non-European countries (Canada, New Zealand, and Australia).

In each country, a sample of around 220 PC physicians completed a questionnaire, except for the smaller countries (Cyprus, Iceland, Luxembourg, and Malta) where this was around 75 PC physicians. In Canada, Belgium, and Spain, larger samples were taken to represent different regions. In most countries, a random sample of PC physicians was invited to participate. Where no national sampling frame was available, alternatives were sought as close as possible to a random sample. Per practice, only one PC physician participated (Groenewegen *et al*., 2016). Sampled PC physicians included specialists in family medicine/general practice for all countries. In some countries, such as Turkey, general physicians without specialty training were also included.

In the practice of each participating PC physician, a trained fieldworker invited consecutive patients entering the waiting room to complete a questionnaire, until 10 questionnaires were administered. Nine patients completed a questionnaire about their experiences with the consultation just finished, while one patient completed a questionnaire about what s/he considered important regarding the care of PC physicians. Apart from their experience, the patient questionnaire also contained some questions related to equity (eg, postponement of care) and use of an emergency department. The PC physician questionnaire consisted of questions on the context of the practice, the human resources and equipment, the employment status of the physician and the structure of the practice, the usual care processes, and the physician activity profiles. The questions were derived from other validated questionnaires. Details of the study design and the development of the questionnaires can be found elsewhere (Schäfer *et al*., 2011; 2013). Ethical review was conducted in accordance with the legal requirements in each country (Pavlic et al., 2015).

### Data analyses

The general approach to data analysis is briefly described here. Details on the variables used and the analysis applied can be found in the corresponding published papers. As the QUALICOPC study has a multilevel design, including countries, PC physician practices, and patients as levels, statistical multilevel models for hierarchically structured data were applied. This allowed variation to be ascribed to each level and to analyse the relations between characteristics at the various levels and outcomes (Schäfer, 2016). Scale scores were constructed through multilevel latent class models. A number of papers used additional data sources, in particular, from the PHAMEU study (Kringos *et al*., 2013) and OECD data on avoidable hospitalisation (OECD, 2013).

## Results

### What are the outcomes of PC?

In this section, we describe the outcomes of PC in terms of patients’ ability to cope better with their health problems, patients’ experiences with the care process in relation to what they find important, equity, and efficient use of care.

Three-quarters of the patients (74%) who visited a PC physician were able to cope better with their health problems following the consultation, and only 7% felt this was not the case. Of the remainder of the patients, 15% indicated that they did not know whether they were able to cope better with their illness and 4% did not answer this question. Swedish patients reported the lowest level of being able to cope better following PC consultations (56%); those from New Zealand noted the highest rates (90%).

**Figure 1.**
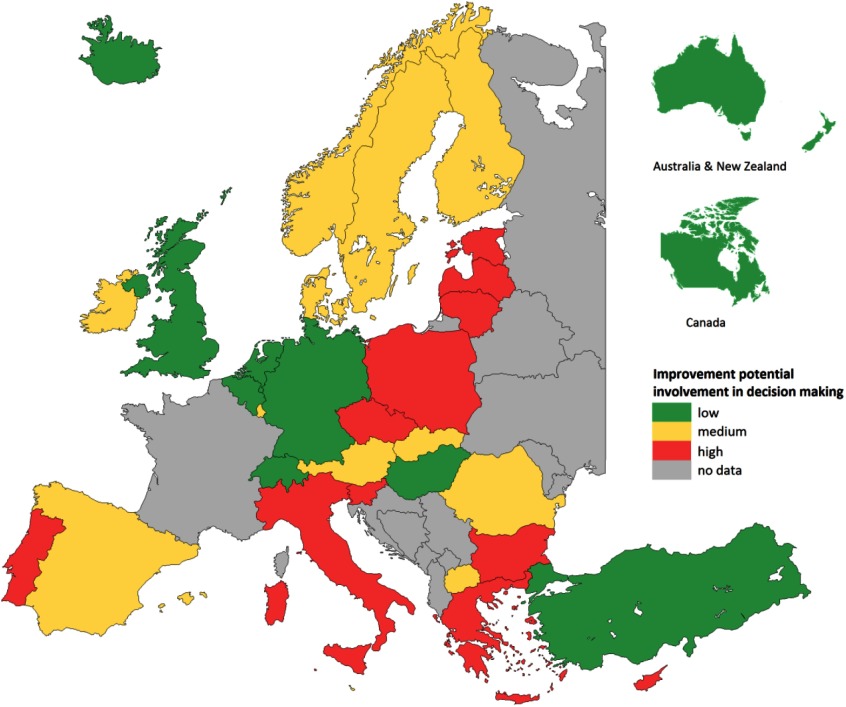
Patient-reported improvement potential for shared involvement in decision making by country

**Source:** QUALICOPC, based on Schäfer *et al.*^2015^) (20).

Patients also reported their *experiences* with doctor–patient communication, accessibility, continuity of care, comprehensiveness, and patient involvement in decision making about the treatment plan. By combining negative experiences with the importance attached to them, we calculated the patient-perceived improvement potential for each country. Based on the range of the scores, the outcomes were divided into three groups (low, medium, and high) (Schäfer *et al*., 2015). Figure 1 shows the improvement potential for patient involvement in decision making about treatment.

PC physicians’ communication showed little potential for improvement in all countries. However, there was a widespread need, in two-thirds of the countries, to have more comprehensive consultations, meaning that multiple problems can be discussed during the consultation, including nonmedical problems.

We found various sources of *inequity* in patient experiences. Migrants, in particular, those of the first generation, were less satisfied, experienced poorer access, and experienced worse communication with their PC physician and less continuity of care (Hanssens *et al*., 2016). Despite high overall satisfaction (92% of the European respondents were satisfied), first-generation migrants, women, and low-income groups were less satisfied with their PC physician (Detollenaere *et al*., 2018). Of all survey respondents, 8.5% indicated that they had postponed care for financial reasons in the previous 12 months. This was even higher for lowincome people. Financially driven postponement of care varied widely between countries, with highest rates in Romania (24%) and lowest rates in Austria (0.7%) (Detollenaere et al., 2016).

**Figure 2.**
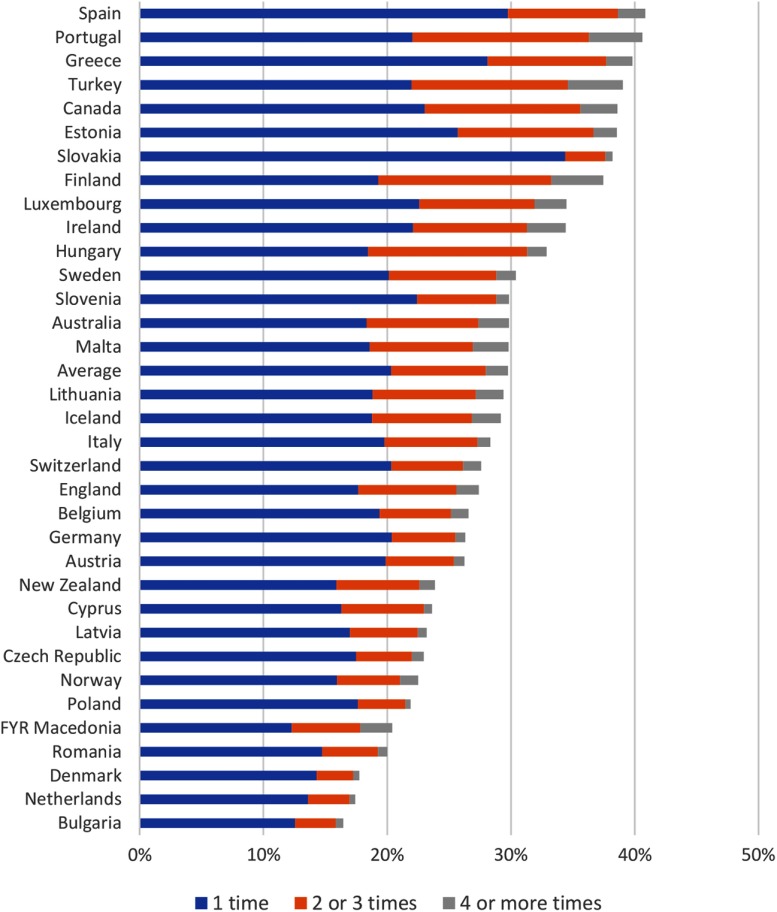
Patient-reported visits to the Emergency Department during the past year by country

**Source:** QUALICOPC, based on Van den Berg *et al.* (2016).

Low (perceived) access to PC may lead to *inefficient use* of care. On average, 29.4% of the patients visited an emergency department in the past 12 months, with substantial variation between countries (Figure 2). Even though ED visits in itself cannot be seen as inefficient use of care, the patients’ responses on main reason for their ED visit pointed towards inefficiency due to problems in PC access: one-quarter indicated that the reason for their ED visit was that there was no PC physician available, whereas only one-third of the ED attenders indicated that they had a problem that could not be dealt by a PC physician (van den Berg *et al*., 2016).

### How do PC processes help to achieve these outcomes?

**Figure 3.**
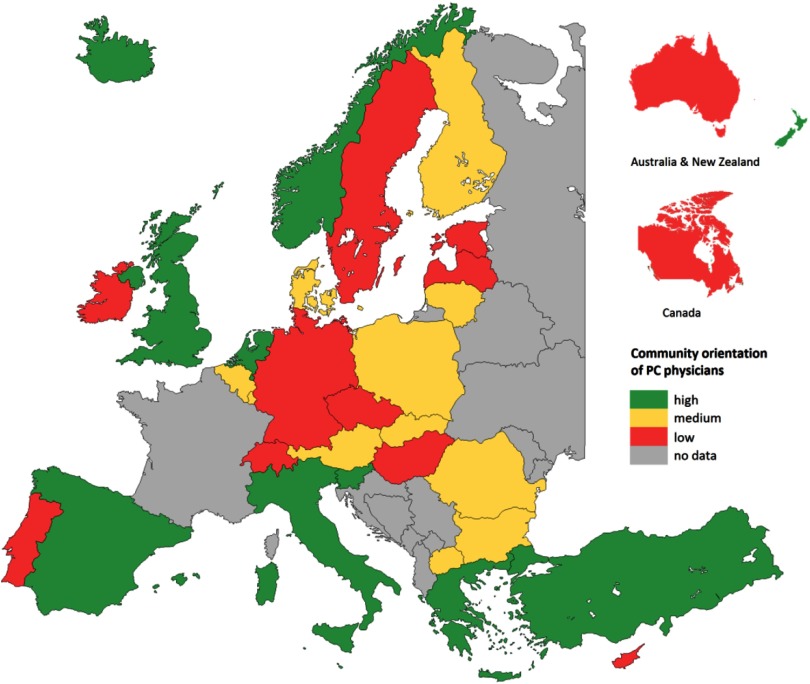
Community orientation of primary care physicians by country

**Source:** QUALICOPC, based on Vermeulen *et al.* (2018).

We start this subsection by describing the care processes and subsequently relating them to the outcomes of PC described in the previous subsection. PC processes in terms of continuity, coordination, comprehensiveness, and community orientation, as reported by PC physicians, vary considerably within and between countries. Community orientation showed the most variation between physicians (Pavlic *et al*., 2015). Community orientation was measured through three questions about the PC physicians’ pro-activity, for example, when confronted with frequent respiratory problems in patients living near factories (Figure 3). Our study showed that PC physicians with a stronger community orientation also provided a broader range of services, in particular preventive services, and more frequently used their medical record system to produce overviews of their practice population (Vermeulen *et al*., 2018).

Currently, there is a near-universal adoption of computers in PC practices. However, in particular, the Southern, Central, and Eastern European countries had low percentages of PC physicians using a computer for clinical functions, such as prescribing and reviewing patients’ medications (De Rosis and Seghieri, 2015). Moreover, PC physicians were asked whether they used their medical record system to list a selection of patients based on age, diagnosis, or risk. This varied from only 3% in Luxembourg to nearly 100% in the Netherlands. To coordinate care with specialists, two-thirds of the PC physicians always send letters to a specialist when they refer a patient; however, less than 40% of PC physicians indicated that they receive feedback communications when their patients are being treated by other medical specialists (Scaioli, Under review).

The comprehensiveness of the services of PC was measured as the range of services they provide. Services provided by PC physicians mostly focused on the treatment of chronic diseases, and the involvement in prevention services in all countries was low (Schäfer *et al*., 2016a).

PC processes are associated with experiences, equity, as well as ED visits. Patients experienced better accessibility, continuity of care, and more involvement in decision making when PC physicians provided more comprehensive care. The comprehensiveness of care mostly explained the variation between countries in patient-perceived accessibility and continuity of care (Schafer *et al*., 2018). More comprehensive, continuous, and accessible PC at the country level was also associated with higher patient satisfaction (Detollenaere *et al*., 2016; 2018). In addition, more comprehensive and accessible PC in a country was associated with less financially driven postponement of PC (Detollenaere *et al*., 2016; 2018). Patients reported fewer visits to an ED when they experienced better access to their PC physician. Moreover, patients with a usual doctor who knows them personally were less likely to attend an ED (van den Berg *et al*., 2016).

The association between avoidable diabetes-related hospitalisation and PC processes was studied because it is an important health problem and associated with high health service utilisation and costs. Diabetes is also a condition for which continuity of care and good coordination in PC can prevent hospitalisation. Lower rates of avoidable diabetes-related hospitalisation were associated with long-term continuity, in terms of a long-term relationship between PC physicians and patients (Van Loenen *et al*., 2016). Countries where PC physicians provided more comprehensive PC services had lower admission rates for long-term complications of diabetes. However, we also found that better access to PC, more comprehensive services, and more medical equipment in PC practices were associated with higher rates of hospital admissions for uncontrolled diabetes (Van Loenen *et al*., 2016). Hospital bed supply in a country turned out to be a major confounder.

### How is the structure of PC practices related to PC processes and outcomes?

In the two previous subsections, the focus was on the outcomes of PC for patients and the care processes in the PC practices. As a next step, we relate these to structural aspects of the PC physicians and their practices.

**Figure 4.**
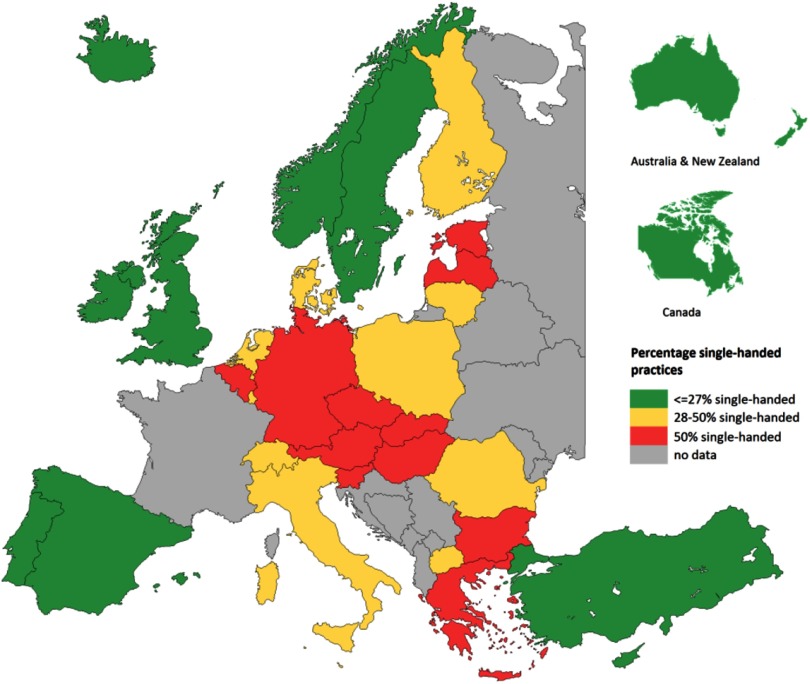
Percent single-handed practices

**Source:** QUALICOPC.

PC physicians are usually paid through a mix of fee-for-service, capitation payments, salary, and pay for performance. Capitation payment is a part of the income of 40% of the PC physicians but absent in, for example, Australia. Moreover, one-third of PC physicians are in salaried service. In some countries, such as Portugal and Spain, almost all PC physicians (95%) are in salaried service, whereas in other countries, they are mainly self-employed (Denmark and Austria). Single-handed practices are common in some countries, such as Slovakia, whereas in Portugal, all PC physicians work in shared practices (Figure 4).

Analyses of the data showed associations between outcomes and processes in PC and these structural aspects. Patient experiences regarding the responsiveness of their PC physician were more positive when PC physicians were paid through capitation (Murante *et al*., 2017). Self-employed PC physicians provided a broader range of services, but not preventive services. In addition, self-employed PC physicians were more community oriented (Schäfer *et al*., 2016b; Vermeulen *et al*., 2018).

Monodisciplinary co-location (PC physicians are co-located with other PC physicians) and multidisciplinary co-location (co-location of PC physicians with at least one other health or social care professional) are related to a provision of more technical procedures, more collaboration between the PC physician and other PC professionals, and coordination with secondary care (Bonciani *et al*., 2018). A separate analysis for Canada and New Zealand showed that multidisciplinary co-location was associated with increased involvement in disease management programmes and an extended role of nurses (Rumball-Smith *et al*., 2014). This may explain our finding that having more professionals working in PC practices in a country was associated with lower admission rates for long-term complications of diabetes (Van Loenen *et al*., 2016). However, multidisciplinary co-location was also associated with less positive patient experiences, especially in countries with a weak national PC structure (Bonciani *et al*., 2018).

Apart from the structural aspects of the practice, the context of PC practices is also related to care processes. PC physicians in rural areas provided a broader range of services, but not preventive services (Schäfer *et al*., 2016b). PC physicians with a relatively high share of people from ethnic minorities in their practice were more community oriented (Vermeulen *et al*., 2018). In addition, practices with a relatively high share of socially disadvantaged patients and/or ethnic minorities also have more different professionals working together in PC (Groenewegen *et al*., 2015).

### How does the national structure of PC facilitate PC processes, structures, and outcomes?

In this final subsection of the results, we discuss the structure of PC as a characteristic of countries and present the results on how this structure relates to the outcomes reported by patients, the care processes, and the structural aspects of the PC practices. The overall structure of PC encompasses three dimensions: governance of PC, the economic conditions of PC, and the workforce development in PC (Kringos *et al*., 2010; 2013). We discuss associations with the overall structure as well as these separate dimensions. Countries that represent a relatively strong PC structure include, for example, the United Kingdom, the Netherlands, and Spain, whereas Cyprus, Luxembourg, and Iceland have a relatively weak structure. An overall stronger structure of PC was related to better patient experiences of continuity and comprehensiveness of care. However, no association between the strength of the overall structure of PC and postponement for financial reasons was found (Detollenaere *et al*., 2016; 2017). In countries with stronger PC governance, patients perceived better continuity of PC. In countries where financial and economic conditions for PC are better, patients reported less room for improvement (Schäfer et al., 2015). In countries with a more pro-PC workforce development, there was more variety in professionals within PC practices (Groenewegen *et al*., 2015). Community orientation was more frequent in health care systems with a list system (Vermeulen *et al*., 2018).

National health expenditures were also related to some aspects of PC outcomes, processes, and practice structures. In countries that spend more on health services, patients reported better experiences in terms of more respectful treatment and patient involvement in the consultation (Murante *et al*., 2017). Higher health expenditures were also positively related to more continuity and more comprehensive processes of care and to computer use (De Rosis and Seghieri, 2015; Pavlic *et al*., 2015).

## Discussion

### Main results

Thirty-five years after the Alma Ata Declaration, the QUALICOPC study collected the information on patients, physicians, and health care systems to analyse the state of PC in 34 (mainly European) countries. After their PC physician consultation, most patients felt that they could cope better with their health problems. Still, specific experiences were not always positive. PC physicians improve consultations mostly by asking patients more frequently about other problems, including psychosocial problems. Patient experiences were more positive when their PC physician provided a broader range of services and when PC physicians were payed through capitation. Experiences were also better when the national structure of PC was stronger and when health care spending was higher.

Equity in access and treatment is an important aim of PC. We found less positive experiences among patients with a migration background and more postponement of PC visits for financial reasons among people on lower incomes. Comprehensive and accessible care is related to more equity. However, we found no relationship between the strength of the national PC structure and patient-level data about the postponement of care for financial reasons. A longitudinal, continuous relationship between PC physicians and patients, a traditional cornerstone of PC, pays off in that those patients are less likely to attend the ED. In countries with more continuous PC, there are fewer avoidable diabetes-related hospitalisations.

### Evidence before this study

Previous research, both international comparative and within the United States, has provided evidence on the benefits of strong PC in terms of aggregated health outcomes and costs of health care (Macinko *et al*., 2003; Health Council of the Netherlands, 2004; Starfield *et al*., 2005). Cost-related studies show that the association between strong PC and health care costs may have changed over time (Kringos *et al*., 2013).

So far, the perspective of patients was lacking, as was insight into the active ingredients of PC in producing favourable outcomes. Evidence on the relationship between PC characteristics and equity was an important gap. The countries included in the QUALICOPC study provided a rich variation in organisation and provision of PC.

### Added value (including strengths and weaknesses)

The added value of our study is that it provides a comprehensive overview on a wide variety of PC processes, practice characteristics, and outcomes for many countries. The direct link between data collected from patients to data collected from the PC physicians they visited enabled a detailed analysis of patient experiences, care processes, and structure and organisation of PC practices. Using statistical multilevel models, we were able to distinguish variation at different levels. We opened the black box of PC and showed the associations between experiences of patients, care processes, structural aspects of PC practices, and national characteristics.

The study also has limitations. The processes at the PC practice level are based on self-reports, and answers may thus be influenced by social desirability. We have not studied health outcomes at the individual level which would require a different study design. The analysis of avoidable hospitalisation did not link the hospitalisations of individual patients to patient questionnaires. As such, there is a risk of an ecological fallacy for these analyses. The study did not include other medical specialists who also provide directly accessible care in some countries. No data were collected from other PC professionals, such as nurses. The design of the study was such that only patients who visited a PC practice were included. The advantage of this is that patients could report on their experience with a recent consultation. The downside is that patients who do not have access to a PC practice were excluded. This implies a potential overestimation of patient-reported accessibility and equity in access in our results.

Response rates were low in several countries, although the samples of PC physicians were representative by age and gender in most countries (Parkinson et al., 2015; Wong *et al*., 2015; Groenewegen *et al*., 2016). Finally, we used a cross-sectional design, and therefore, the associations that we have found cannot be interpreted causally.

### Implications for policy makers and practice

Patient experiences can be improved by PC practices by changing the care processes and practice organisation and by policy makers at the national (or regional) level by improving the conditions that could facilitate strong PC.

However, making health systems more PC oriented requires a comprehensive vision on the health system as a whole. Supporting strong PC is likely to fail when secondary care providers have incentives to increase production in order to keep using available capacity. The study on avoidable diabetes-related hospitalisations showed the importance of hospital bed supply in a country. On the patient side, incentives, such as low cost sharing for PC or increased cost sharing for secondary care, could be brought in place to nudge them towards using PC instead of going directly to an ED or to a secondary care.

The organisation of PC should be tuned into the needs of communities. These needs may differ between countries and local communities, and they may change over time. It is reassuring that PC physicians in many countries have adapted to the increased need for treatment of (multiple) chronic conditions, but preventive activities and community orientation are still limited. The needs of communities can be assessed more easily when PC practices have a fixed patient list or defined catchment area.

Changing care provision and organisation is a huge challenge. For instance, implementation of a patient list or registry system in a country without this tradition is very difficult. Countries in Central and Eastern Europe that have oriented their policies towards strengthening PC over the past twenty-five years still show variable outcomes in achieving their aims. Based on studies including QUALICOPC, we now know more about *what* should be done to strengthen PC; however, there still is a gap between this increased knowledge and *how* changes to strengthen PC should be implemented. Professionals in different positions as well as patients may resist the changes needed to implement stronger PC.

### Impacts of the study until now

It is too early to assess the current impacts of the study. However, in many countries, the data have been used to analyse the national situation and many results have been published in the national languages. This shows the need for this type of information and that there are ways to disseminate the results to policy makers and practitioners in different countries. In the Netherlands, for example, an additional analysis of workload of Dutch PC physicians was made. This analysis plays a role in current discussions about the desirability of decreasing patient list sizes.

The OECD is currently working on an international study on patients with chronic conditions as part of the Patient-Reported Indicators Survey initiative (OECD, 2018). The experience built with the QUALICOPC study served as input in the development of the design.

## Conclusion

Although much has changed over the past 40 years, essential aspects of the Alma Ata Declaration have not lost their urgency, as also emphasised through the 2018 Astana Declaration (Declaration of Astana, 2018). PC is strong and successful in many countries, but results of the QUALICOPC study also show that there is room for improvement in many health systems. Strong PC pays off in terms of patient experiences, equity, and efficiency. Successful investments can only be done when underpinned by evidence, such as provided by this study. As the context and organisation of PC are changing continuously, there is a permanent need to monitor these changes and to evaluate the effects on health outcomes, patient experiences, equity, and efficiency.
